# Tracing Selenium Uptake in Wheat Leaves via Liposome-Mediated Delivery: A Confocal Microscopy and Synchrotron Micro-X-ray Fluorescence Insight

**DOI:** 10.1021/acsagscitech.5c01076

**Published:** 2026-03-05

**Authors:** Marcia Viltres-Portales, María-Jesús Sánchez-Martín, Roberto Boada, Mercè Llugany, Manuel Valiente

**Affiliations:** † GTS Research Group, Department of Chemistry, Faculty of Science, 16719 Universitat Autònoma de Barcelona, 08193 Bellaterra, Spain; ‡ GTS Research Group, Department of Chemistry, Faculty of Science, Universitat Autònoma de Barcelona, 08193 Bellaterra, Spain; § GTS Research Group, Department of Chemistry, Faculty of Science, Universitat Autònoma de Barcelona, 08193 Bellaterra, Spain; ∥ Plant Physiology Group (BABVE), Faculty of Biosciences, Universitat Autònoma de Barcelona, 08193 Bellaterra, Spain; ⊥ GTS Research Group, Department of Chemistry, Faculty of Science, Universitat Autònoma de Barcelona, 08193 Bellaterra, Spain

**Keywords:** wheat, selenium, liposomes, confocal fluorescence microscopy, synchrotron micro-X-ray fluorescence

## Abstract

The integration of nanoencapsulation techniques with foliar application presents a promising approach to enhance selenium (Se) biofortification in agriculture. This study examined the foliar uptake of liposome-encapsulated Se in wheat leaves (*Triticum aestivum*) using synchrotron-based *micro*-X-ray fluorescence (μ-XRF) and confocal microscopy. μ-XRF mapping showed Se accumulation at leaf edges after 24 h, suggesting initial uptake via stomata, while free Se was absorbed and transported more rapidly, highlighting the slow-release effect provided by liposomal encapsulation, longer than the analyzed time. No immediate translocation of Se to the stem was observed, suggesting that more time is required for this internal movement. *Micro-*X-ray absorption near-edge structure (μ-XANES) speciation analysis demonstrated that Se was metabolized into organic forms within the plant. Finally, confocal fluorescence microscopy confirmed liposome absorption through the plant surface within 24 h, corroborating the μ-XRF findings. These results are crucial for optimizing liposome formulation to maximize Se transfer to edible parts.

## Graphical abstract

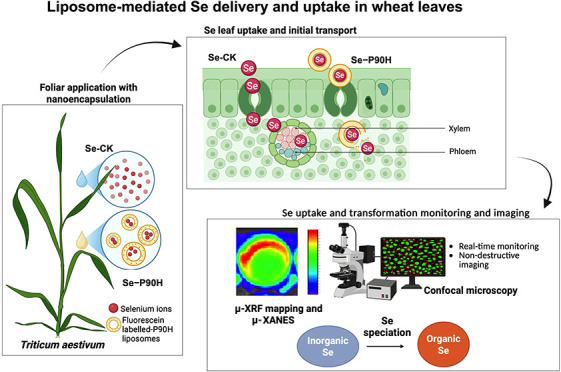

## 1 Introduction

The foliar uptake of nutrients, fertilizers and other agrochemicals is a critical research area in modern agronomy and plant physiology.^1^ Understanding these processes is essential not only for optimizing agricultural practices but also for explaining the fundamental physiological mechanisms that regulate plant nutrition.^2^ Over recent decades, foliar fertilization has gained increasing attention as a complementary strategy to soil fertilization, offering a faster and efficient absorption of essential elements.^3,4^ This approach is particularly relevant under conditions where soil nutrient availability is limited or when an immediate response to nutrient deficiencies is required.^5,6^

A comprehensive understanding of foliar uptake is crucial for developing more efficient and sustainable fertilization strategies.^7^ This includes investigating nutrient absorption through the leaf surface, subsequent translocation within plant tissues, and the key factors influencing uptake efficacy. Among these factors, the physicochemical properties of applied solutions, environmental conditions, and the plant’s physiological status play a decisive role.^3^ Furthermore, advances in this field have the potential to significantly enhance agricultural productivity and crop quality while mitigating the environmental impact associated with the excessive use of conventional fertilizers.^8^

In recent years, nanotechnology has emerged as a promising approach for improving the efficiency of foliar nutrient application.^9^ The encapsulation of different compounds within nanomaterials has demonstrated significant advantages, including enhanced absorption, controlled release, and reduced environmental footprint compared to traditional fertilizers.^10–13^

Among micronutrients of global concern, selenium (Se) has been extensively studied because of its dual role as an essential element for human nutrition and a beneficial element for plants at low doses, contributing to antioxidant defense and stress tolerance.^14^ Current Se nanobiofortification approaches predominantly rely on either ionic Se salts (selenate or selenite) or elemental Se nanoparticles (SeNPs).^15^ Selenium nanoparticles have shown promising results in terms of enhanced bioavailability, reduced phytotoxicity compared to ionic forms, and improved stress resilience in several crops.^16^ However, the agronomic performance of SeNPs remains highly dependent on particle size, crystallinity, surface charge, and synthesis route, which strongly influence their dissolution kinetics, transformation inside plants, and interaction with soil.^17,18^ Importantly, despite increasing reports of low acute toxicity, uncertainties persist regarding the long-term environmental fate, and potential bioaccumulation of nanoparticles in agroecosystems.^19,20^ These unresolved issues currently limit their regulatory acceptance and large-scale agricultural deployment.

In contrast, nanocapsule-based delivery systems, particularly liposomal nanocarriers, represent an emerging and underexplored strategy for selenium biofortification. Liposomes are phospholipid vesicles capable of encapsulating selenium species within a biocompatible bilayer, enabling protection, improved adhesion to leaf surfaces, and controlled release following foliar application.^21^ Unlike inorganic nanoparticles, liposomes are composed of phospholipids generally recognized as safe, biodegradable, and already widely used in food, pharmaceutical, and nutraceutical industries.^9^ Therefore, within the broader landscape of nanobiofortification strategies, selenium-loaded nanoliposomes offer a compelling alternative to selenium nanoparticles by combining efficient nutrient delivery with superior biocompatibility.

Recent studies have shown that nanoparticles such as liposomes and polymeric nanospheres can overcome the physical barriers of the leaf cuticle and stomata, achieve bidirectional transport (leaf-to-root) and improving nutrient bioavailability.^22^ For instance, soy phospholipid-based liposome (∼100 nm) showed a 33% foliar penetration in tomato plants, a significant improvement compared to the 0.1% penetration observed for free nutrients, effectively addressing acute Fe and Mg deficiencies.^23^ Similarly, studies in crops such as *Hordeum vulgare* (barley) has reported up to an 18-fold increase in Cu accumulation in plant tissues using nanofertilizers,^19^ while hydroxyapatite-based formulations enhance phosphorus uptake in acidic soils.^20^ A study by Farshchi et al.^12^ further supports the efficacy of nanoliposomes in the foliar fertilization of sweet basil (*Ocimum basilicum* L.), demonstrating that Fe-liposome treatment significantly enhanced the levels of total Fe and Fe^2+^ ion in plants compared to traditional FeSO_4_-EDTA fertilizers in terms of iron delivery and plant recovery, highlighting their potential to improve both nutritional and qualitative aspects of crop production.

Moreover, liposomal formulations allow coencapsulation of micronutrients with additional bioactive compounds (e.g., antioxidants, phytohormones, or other mineral nutrients), offering multifunctional platforms for synergistic crop nutrition and stress mitigation.^24,25^ However, in general, challenges persist, including the standardization of nanoparticles production protocols, the long-term evaluation of potential toxicity, and the optimization of application parameters such as particle size, concentration, and synchronization with the plant’s phenological stages.

In this context, the ability to visualize the absorption pathways of nanocarriers and their contents is essential for understanding the underlying physiological mechanisms and optimizing their practical application as foliar fertilizers. Advanced imaging techniques provide valuable insights into the absorption and distribution processes within plant tissues. Confocal fluorescence microscopy and synchrotron-based *micro*-X-ray fluorescence (μ-XRF) have emerged as powerful tools for visualizing and quantifying the uptake and distribution pathways of foliar-applied nanocarriers and their loads, considering their high spatial resolution, nondestructive or minimally invasive nature, chemical specificity, accurate nutrient quantification, and complementarity.^26–29^

Confocal fluorescence microscopy has proven particularly effective in revealing how leaf surface characteristics influence nutrient absorption. Studies have shown that factors such as trichome density and stomatal aperture can significantly affect the penetration of foliar-applied nutrients. For instance, high trichomes density may increase leaf hydrophobicity, while stomata can facilitate nutrient uptake. This technique has also been instrumental in visualizing the distribution of nanocarriers internalized into the leaf mesophyll, providing insights into the uptake and translocation of novel agrochemical delivery systems.^28^ In this study, core–shell nanocapsules were applied to tomato leaves, demonstrating that nanocapsules with optimized surface chemistries and sizes were internalized into the mesophyll within 24 h of foliar application. X-ray fluorescence mapping was also employed to track the distribution of encapsulated tracer metals, providing a dual-modality approach for studying nutrient translocation and phloem loading efficiency.

In another study, Kohay et al.^29^ compared the effects of applying layered double hydroxide (LDH) nanoparticles to the adaxial (upper) versus abaxial (lower) surfaces of tomato leaves for foliar delivery of nutrients and genetic material aimed at enhancing plant growth and yield. Confocal microscopy was used to visualize the uptake routes of LDH nanoparticles, allowing for detailed observation of how these particles penetrate leaf tissues. It also facilitated the quantitative analysis of LDH coverage on the leaf surface and stomatal aperture area, contributing to a better understanding of how application methods influence nanoparticle delivery. Furthermore, this technique enabled the analysis of LDH relative localization to the cuticle, providing insights into the distribution and interaction of nanoparticles within the leaf structure.

Synchrotron-based μ-XRF has complemented these findings by offering high-resolution elemental mapping of plant tissues. This method allows for the investigation of the distribution patterns of microelements applied via foliar sprays with high sensitivity, potentially facilitating the development and optimization of foliar fertilizer application techniques.^30^ Recent studies have utilized μ-XRF to compare the transport of different forms of foliar-applied nutrients, such as zinc sulfate and zinc-EDTA, revealing differences in their mobility and efficacy within plant systems.^26^ Arsic et al.^31^ visualized phosphate uptake pathways in phosphorus-deficient barley leaves using bioimaging techniques, including synchrotron-based methods. This research highlighted the potential of foliar phosphorus applications to restore photosynthetic processes by tracing phosphorus distribution within leaf tissues.

A previous study by our group demonstrated a novel use of phosphatidylcholine-based liposomes for Se uptake in wheat plants, achieving 1.5-fold higher efficiency compared to the direct application of an aqueous Se solution.^32^ However, information on the mechanisms responsible for this enhanced uptake and subsequent distribution is still unclear in scientific literature. In the present work, our objective was to provide a proof of concept for the application of synchrotron-based μ-XRF and μ-XANES techniques, combined with confocal fluorescence microscopy to investigate the Se and liposomes uptake, translocation and chemical speciation in wheat tissues over a 24 h period (shortly after foliar application of the nanoliposomal formulation), comparing liposome-encapsulated Se to free Se. Rather than performing a long-term evaluation of selenium biofortification, this work focuses on elucidating the initial behavior of Se at the shoot level.

## 2 Materials and Methods

### 2.1 Reagents

Phospholipon 90H (90% hydrogenated phosphatidylcholine from soybean) was purchased from Lipoid GMBH, Ludwigshafen, Germany; sodium selenite (Na_2_SeO_3_), 2-(*N*-morpholino)­ethanesulfonic acid (MES), calcium chloride (CaCl_2_) and Triton X-100 were purchased from VWR Internationals LLC, Barcelona, Spain; Tissue-Tek O.C.T. was purchased from Sakura, Finetek USA, Inc., Torrance, California; isopentane, seleno-l-methionine (SeMet), seleno-l-cystine (SeCys) and Se-(Methyl)­selenocysteine hydrochloride (MetSeCys) were obtained from Sigma Merck, Schnelldorf, Germany; and sodium selenate was purchased from Acros Organics, Barcelona Spain.

### 2.2 Liposomes Preparation

Phospholipon 90H was used to prepare the Se encapsulated liposomes (hereafter referred to as P90H) following the lipid film hydration protocol and Na_2_SeO_3_ was used for encapsulation, as described in previous work.^16^ The size distribution profile, polydispersity, and zeta potential of the vesicles were analyzed by dynamic light scattering (DLS) (Zetasizer Nano ZS, Malvern Instruments Ltd.). Measurements were carried out at 25 °C with a detector angle of 90° within 24 h of preparation and following sonication for 5 min above 55 °C. Se encapsulation efficiency and loading capacity in P90H liposomes was determined by quantifying nonencapsulated Se in the filtrate following centrifugation through 10 kDa molecular weight cut-off (MWCO) filters and via ICP-MS (X Series 2, Thermo Elemental) according to Peng et al.^33^ and Boelter and Brandelli^34^ (See Supporting Information for equations and characterization results). Liposomes were stored at 4 °C until their application to the plants.

### 2.3 Plant Growth

Wheat plants were grown to study the uptake of Se species contained in the P90H liposomes. Seeds of *Triticum aestivum* L. cv. Bancal (Fitó S.A., Barcelona, Spain) were germinated on moistened wrapped cellulose paper using tap water, under 5 days of darkness followed by 2 days of light. The germinated seedlings were then transferred to a hydroponic system consisting of plastic pots filled with half-strength Hoagland’s nutrient solution, buffered with MES to maintain a stable pH of 6.0 and continuously aerated.^35^ Environmental conditions were controlled as follows: temperature 18–22 °C, relative humidity 50–60%, light intensity 320 μE·m^–2^·s^–1^, and a photoperiod of 16 h light/8 h dark. Hydroponic cultivation system was chosen over conventional soil to allow for precise control of nutrient availability.

### 2.4 Selenium Determination

After 5 weeks of growth, foliar Se treatments were applied to the wheat plants. Plants were treated with either 1 mM Na_2_SeO_3_ encapsulated in liposomes (Se–P90H) or 1 mM Na_2_SeO_3_ aqueous solution used as control (Se-CK). These terms will be used throughout this study. One week after application, the plants in the hydroponic pots were harvested (*n* = 6; three plants per pot, two pots per treatment). Each plant was immersed for 10 min in an ice-cooled 10 mM CaCl_2_ solution to remove any remaining surface nutrients.^35^ Shoots (stem and leaves) were separated from roots. Shoots from Se–P90H treated plants were thoroughly washed with 1% Triton X-100 followed by milli-Q water to remove any unabsorbed Se and liposomes from the leaf surface. Shoots from Se-CK plants were washed only with milli-Q water. Excess water was removed with absorbent paper, and shoots and roots were weighed and stored at −20 °C for further analysis. Frozen samples were lyophilized (Telstar lyoquest, Telstar) for 48 h and ground. Ground, dry plant samples (0.4 g) were digested with a HNO_3_/H_2_O_2_ mixture (9:1, v/v) using a microwave oven (MARS 2, CEM, USA). The total Se concentration in the digested filtrates was analyzed using inductively coupled plasma mass spectroscopy (ICP-MS; Agilent 7900, USA).^32^

### 2.5 Synchrotron-Based μ-X-ray Fluorescence Analysis

μ-XRF provided the elemental distribution and speciation of Se throughout the leaves and stems of the plant after foliar application, in comparison to free Se. For the analysis, the Se-CK and Se–P90H treatments were applied foliarly at 5 weeks of growth to wheat plants, targeting the first half of the second last fully expanded leaf (one leaf below the newest growth) as shown in Figure 1. The reminder of the plant and the hydroponic pot were covered with plastic to prevent the deposition of sprayed particles. Leaves and their corresponding stems were cut at 24 h after application for a short-term absorption study. Tissues were washed as previously described and the samples for the analysis were selected from the posterior zone of the leaf, far from the application site, as represented in Figure 1.

**Figure 1:**
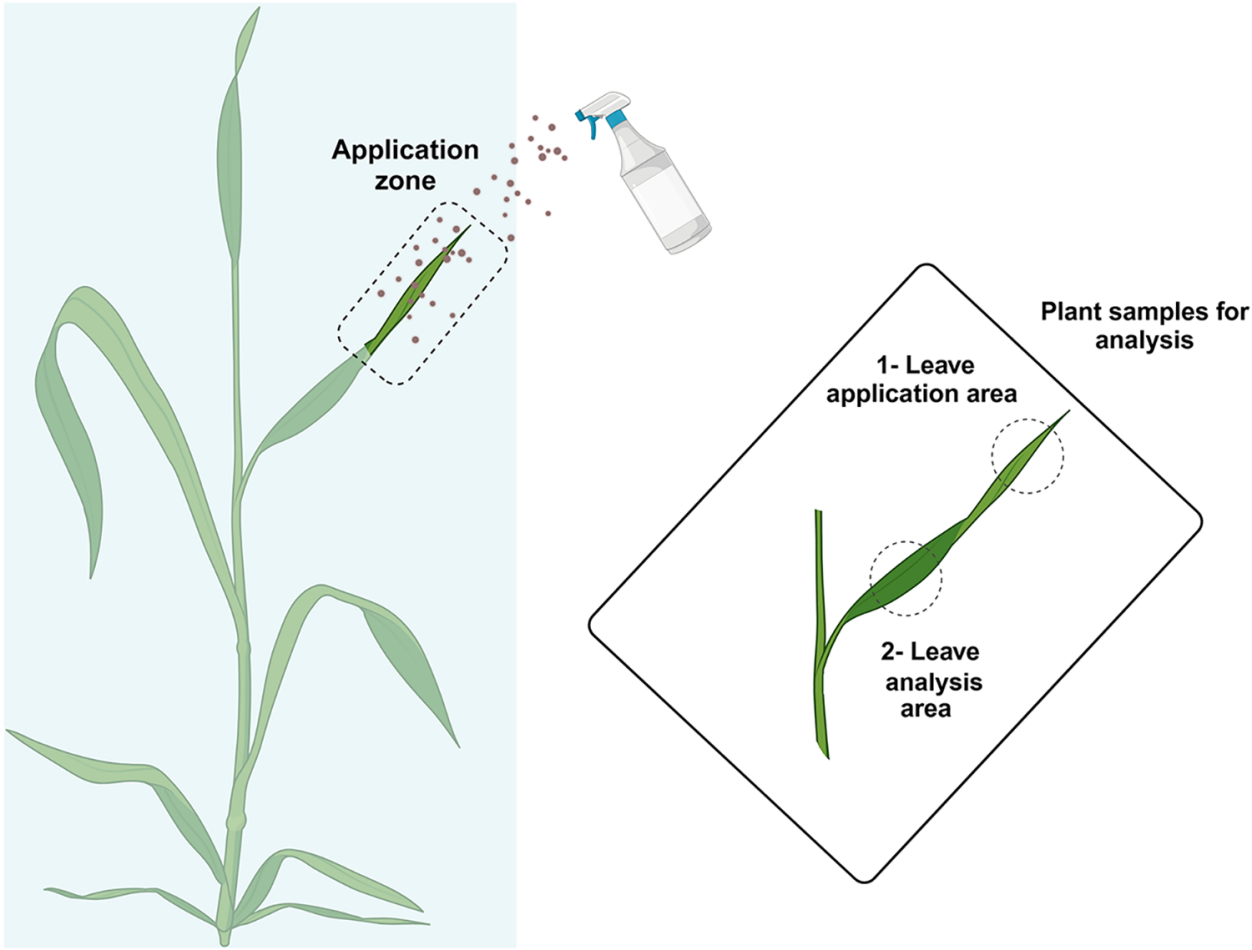
Scheme of the treatment application and sample selection for μ-XRF analysis at Diamond synchrotron.

To obtain thin sections for the μ-XRF measurements, plant samples were immediately frozen upon excision. Samples were embedded in OCT compound within plastic cryomolds (25 mm × 20 mm × 5 mm, Sakura, Finetek USA, Inc.) and rapidly frozen by immersion in precooled isopentane chilled with liquid nitrogen. Thin sections (30 μm) were prepared using a cryotome (Leica CM3050S, Leica Biosystems, Spain), immediately mounted onto sapphire discs and stored at −80 °C until analysis.

μ-XRF mapping and μ-XANES measurements were conducted at the I18 beamline^36^ of the Diamond Light Source (Didcot, U.K.) using a Si(111) double-crystal monochromator and a pair of Kirkpatrick-Baez focusing mirrors that allow beam size adjustment to match experimental requirements. A 4-element silicon drift detector (Vortex) was used to collect the fluorescence signal from the samples. The samples, affixed to the sapphire discs with OCT, were positioned on the aluminum sample holder inside a liquid helium cryostat. Measurements were performed at 5 K to minimize radiation-induced damage. The spatial distribution of Se, K, Mn, and Zn in plant tissues was determined from the μ-XRF maps acquired using an excitation energy of 13,450 eV above the Se–K edge. The beam size was focused to 3 μm and maps were acquired using a step size of 10 μm. The acquisition time was set to 0.05 s per point. μ-XRF maps were processed using DAWN software^37^ and multicolor maps were generated using the RGB mixer tool, which allows for the combination of μ-XRF maps from different elements. Intensity levels were adjusted to optimize elemental visualization.

To account for spatial heterogeneity, μ-XANES spectra at the Se K-edge were acquired in fluorescence mode at three different points (Figure 3A) of each region where Se was detected in the plant tissues (leaves and stems) and merged. Spectral normalization and speciation analysis were performed using the Athena program within the Demeter software package.^38^ Linear combination fitting (LCF) analysis was conducted using spectra of sodium selenite, sodium selenate, SeMet, SeCys and MetSeCys as references.

### 2.6 Confocal Microscopy Study

Fluorescent labeled liposomes were prepared by incorporating fluorescein^23^ as fluorescein diacetate (FDA; Sigma-Aldrich, Germany) at a final concentration of 1 mM into the lipid solution dissolved in CHCl_3_ during the thin-film formation process. After solvent evaporation, the lipid film containing FDA was hydrated with aqueous buffer to form the vesicles, which were subsequently sonicated to reduce vesicle size and homogenize the suspension. Free and nonencapsulated fluorescein was removed by centrifugation using 10 kDa MWCO centrifugal concentrators and then resuspended in MES buffer to the desired concentration via sonication. Purified fluorescently labeled liposomes were used for further analysis. For leaf application, five drops (20 μL per drop) of the fluorescently labeled liposome suspension were placed at the center of the adaxial surface of the last fully expanded leaf. After application, plants were kept in complete darkness for 24 h under the same controlled growth conditions (three replicates plus one untreated control). Following this period, treated leaves were excised and washed with 50% (v/v) acetone followed by milli-Q water to remove any residual surface-associated fluorescence.

Liposome penetration was assessed directly on two zones of the excised leaf tissue, which was cut into rectangular sections (1.5 cm × 2 cm) mounted on microscope slides the edge zone and the central cut zone, for both control and treated samples (Figure 4). Confocal laser scanning microscopy experiment was performed using an Axio Observer 7 microscopy (Zeiss LSM 980 Airyscan 2) following the methodology described by Zhu et al.,^39^ with modifications to adapt the imaging conditions to the present samples. Z-stack images were acquired by sequential scanning parallel to the leaf surface using a 20× objective lens, with a step size of 1 μm between optical sections. Fluorescein was excited at 495 nm, and emission was collected between 491 and 562 nm, while chlorophyll autofluorescence was detected in the red channel. The analysis was performed immediately after excision to prevent tissue dehydration. Images were processed using Zen software.

## 3 Results and Discussion

### 3.1 μ-XRF Mapping of Se Uptake and μ-XANES Speciation

μ-XRF measurements allow the elemental mapping of plant tissues, enabling the direct observation of Se distribution across different parts of the wheat plant. The μ-XRF maps presented in Figure 2 show Se accumulation in the posterior leaf area (see Figure 1) after 24 h following application. Since μ-XRF simultaneously provides information on the spatial distribution of several elements accumulated in the plants, in our study, RGB maps of the different analyzed elements were used to help in the visualization of the distribution patterns and assess their colocalization.^40^ As shown in Figure 2, K was distributed throughout the leaf, revealing the internal structure, while Se was mainly located along the outer margins of the leaf. See Supporting Information Figure S3 for all obtained maps.

**Figure 2:**
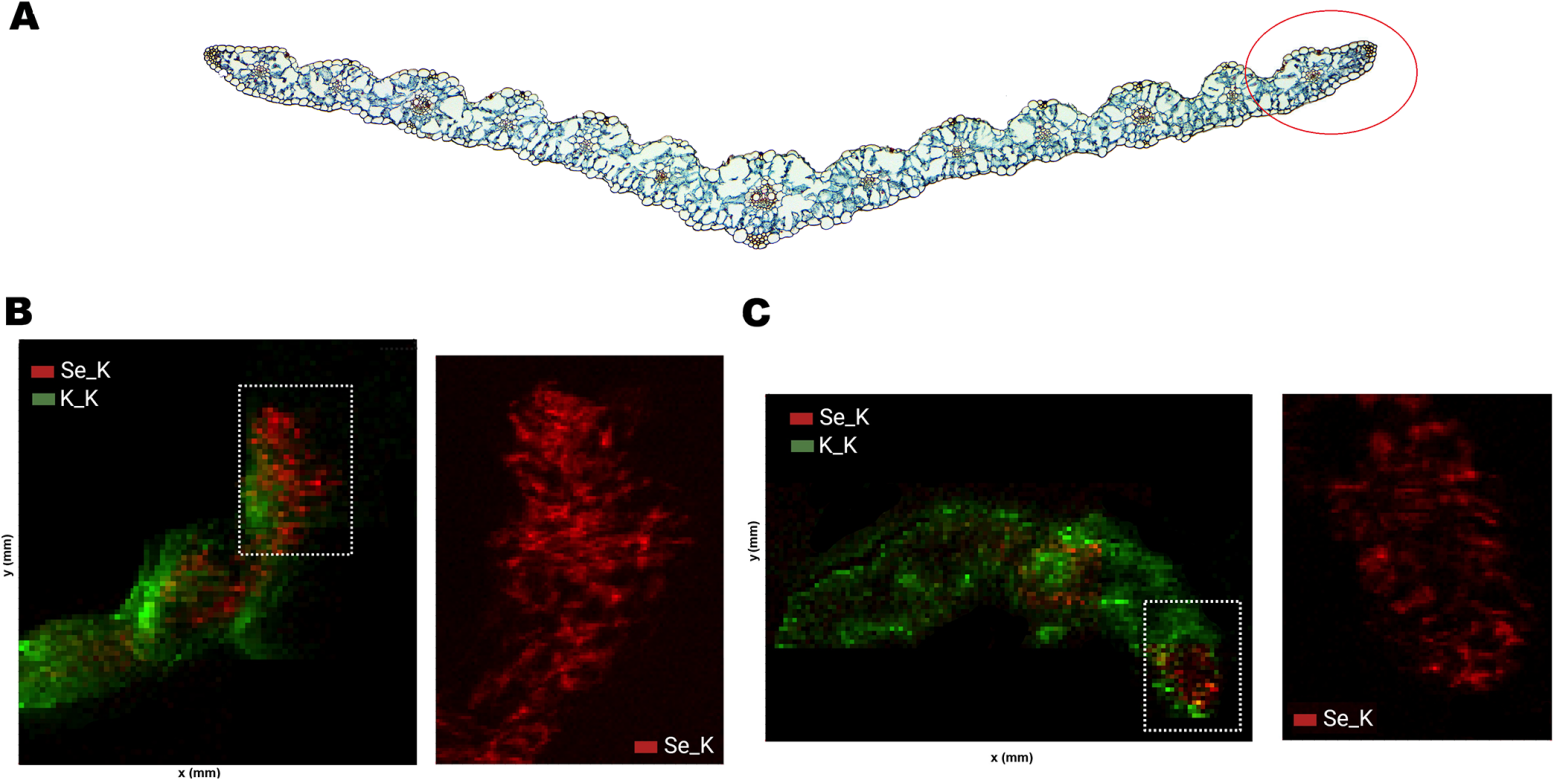
Synchrotron μ-XRF mapping results: Schematic representation of the approximate analyzed area in the posterior zone of a wheat (*Triticum*) leaf cross-section (A). Elemental maps of wheat leaf cross sections 24 h after exposure to Se-CK (B) and to Se–P90H (C). The bottom panels (from left to right) show two-color merged maps of elemental Se (red) and K (green), followed by an enlarged detailed map of Se corresponding to the white highlighted area.

X-ray fluorescence images also revealed a nonhomogeneous Se distribution in wheat leaves, extending from the adaxial to the abaxial epidermis. Since the Se_K maps were adjusted to the same relative scale, Se distribution in the Se-CK samples (Figure 2A) appeared more extensive and exhibited higher fluorescence intensity compared to Se–P90H treated plants (Figure 2B), suggesting that Se encapsulated within P90H liposomes requires more time for uptake, release and translocation into plant cells.

Based on ICP-MS analysis, the total Se concentration in wheat shoots was 1.6 ± 0.2 μg Se·g^–1^ DW for Se-CK and 2.5 ± 0.4 μg Se·g^–1^ DW for Se–P90H treated plants 1 week after application, indicating that the liposome encapsulation enhances total Se uptake by the plants.

Consistent with these findings, some studies have demonstrated that encapsulated compounds are gradually released from their carriers and absorbed by plant leaves over an extended period, ensuring a sustained release and enhancing uptake efficiency. For instance, nanobiofertilizer capsules showed sustained cumulative NPK release over 30 days, reaching 33.2, 47.8, and 68.3% for different nutrients, indicating a slower release profile compared to free nutrients.^22^ In the case of liposomes composed of plant-derived lipids, uptake and release of their active ingredients over time revealed that 24 h after application, 7–19% of the applied dose was absorbed, increasing to 27–33% after 72 h.^9^ In this same study, liposomes loaded with europium (a tracer molecule) exhibited declining concentrations with increasing distance from the application point, suggesting a gradual distribution within the plant over 24–72 h.

μ-XANES was used to investigate the chemical transformation of applied Se in the regions where this element was detected. Figure 3B shows the comparison for the Se-CK treatment with Se references as a representative case of study, since spectra from Se–P90H samples were only qualitatively informative (see Supporting Information, Figure S2).

**Figure 3:**
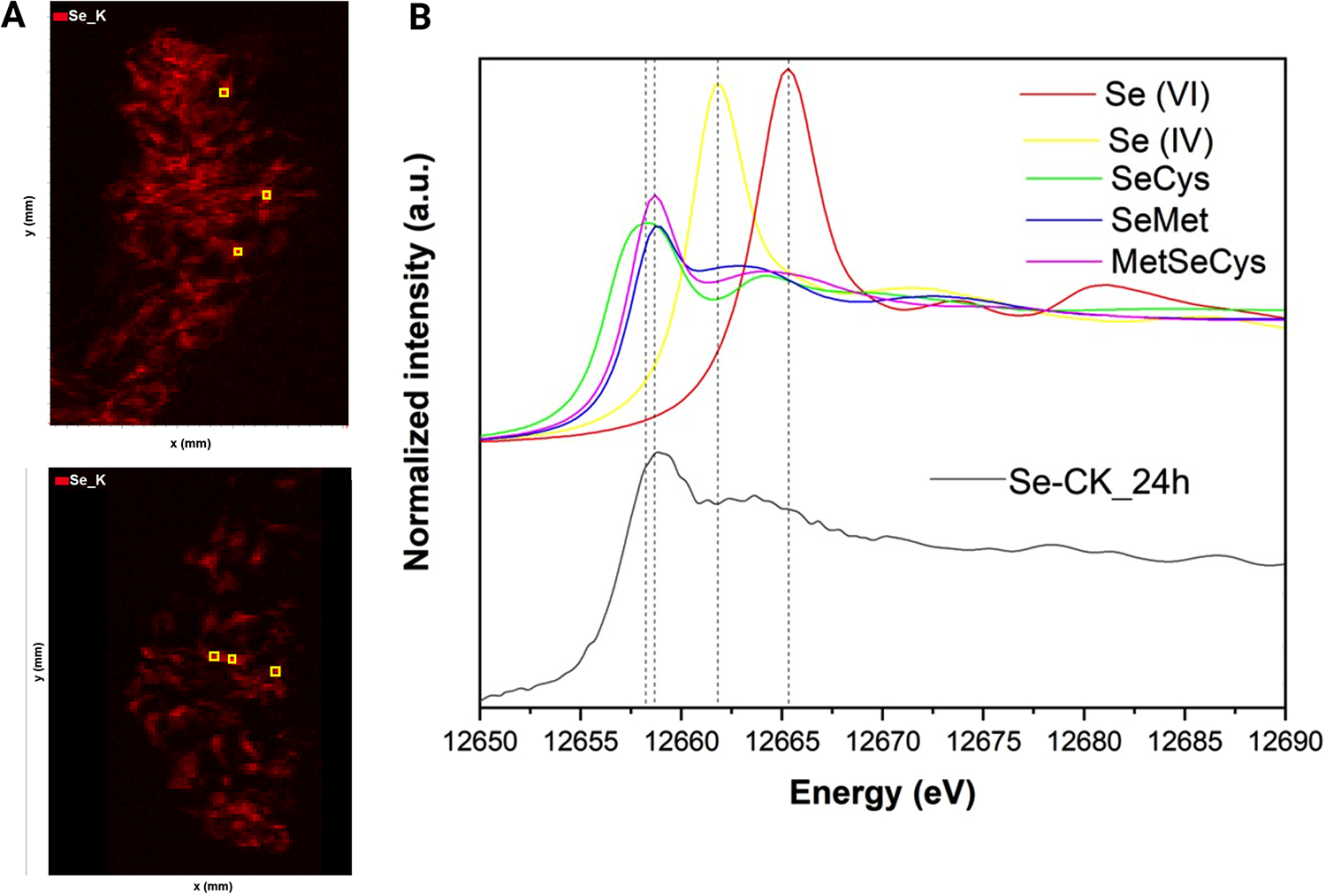
Combined synchrotron μ-XRF and μ-XANES: μ-XRF images of Se_K for the outer cross-section area of two different leaves exposed to Se-CK (A) and normalized Se K-edge XANES of Se references and the merge of the μ-XANES recorded at yellow sites highlighted in A (B).

The μ-XANES spectral profile of Se-CK did not resemble the one of inorganic selenite species used for the biofortification application but that of the organic Se species, as the spectrum is characterized by a white line at 12657 eV, like the organic references containing C–Se–C (e.g., SeMet and MetSeCys) or C–Se–Se-C (e.g., SeCys) bonds. The results from the LCF analysis (Table S2) confirmed that Se was present as organic species, that could be SeMet (72.6%), SeCys (20.6%) and MetSeCys (11.7%) according to the references. These results agree with current findings emphasizing that SeMet is generally the principal organic form found in Se-enriched staple crops.^41^ The rapid and efficient conversion of inorganic Se to organic species is notable, given that Se was foliarly applied as selenite (SeIV), which can be readily reduced to selenide and subsequently incorporated into selenoamino acids via the sulfur assimilation pathway.^42,43^ However, other selenium species, such as selenate (SeVI), require additional enzymatic reduction steps prior to assimilation and are therefore generally metabolized less directly.^42,44^ This finding highlights the high metabolic capacity of wheat leaves to assimilate Se following foliar application.

### 3.2 Confocal Fluorescence Imaging of Wheat Leaf to Study Liposome Penetration

Confocal fluorescence images were obtained from two sections of the treated wheat leaves, corresponding to the near-edge zone and the cut zone (Figure 4-scheme), in both control and treated samples. Two different fluorescence signals were monitored: fluorescein probe and chlorophylls for the visualization of internal leaf structures. Images were acquired at the same depths from the adaxial surface of the leaf. In the control samples, only red structures were observed (Figure 4a,b), corresponding to chloroplasts within plant cells. In the case of the treated samples (Figure 4c,d), green fluorescence was detected around both the outer and inner edges of the leaf sections, indicating the presence of fluorescein. A more detailed 3D image (Figure 5) revealed oval-shaped fluorescein structures inside the plant surrounding the stomata.

**Figure 4:**
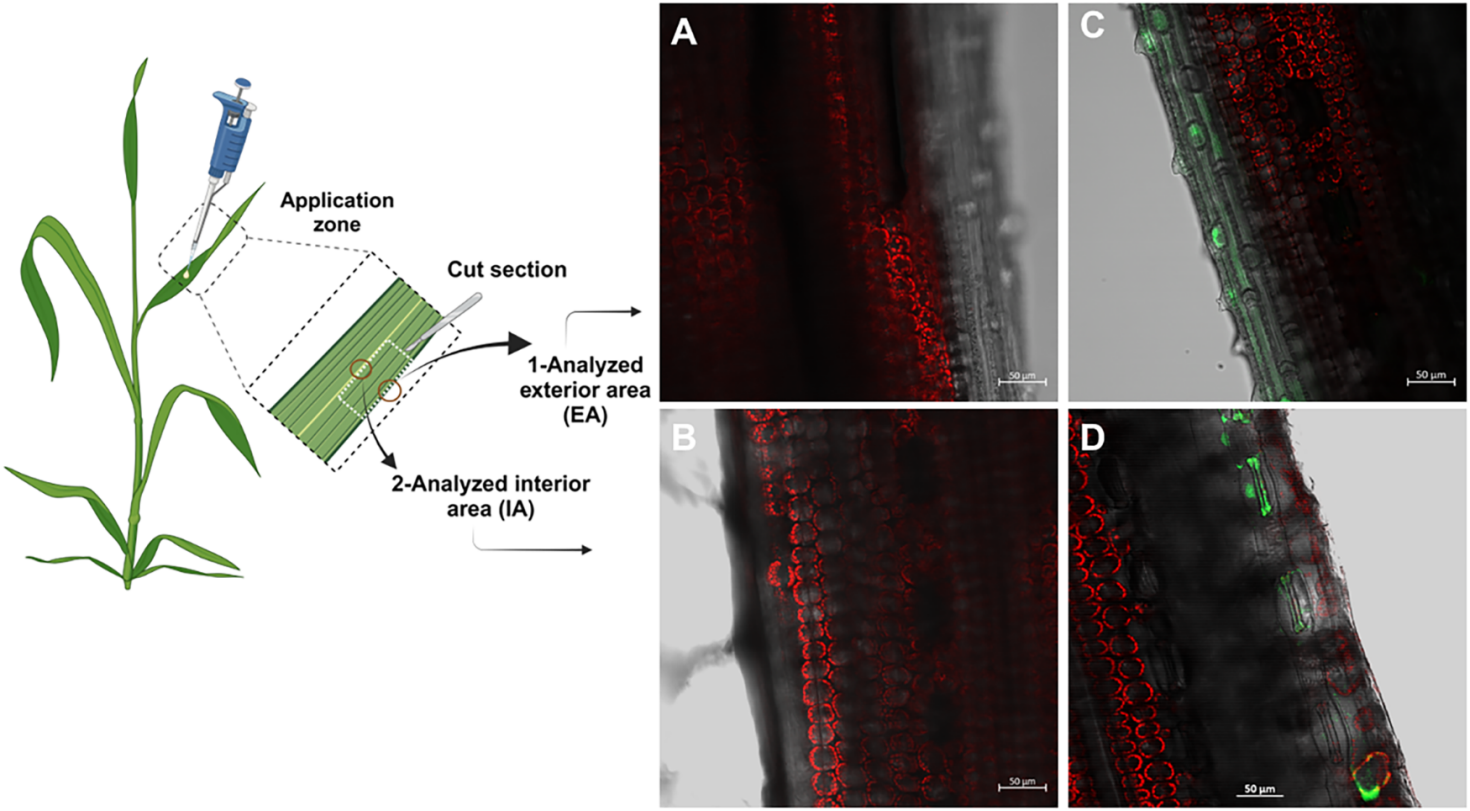
Confocal fluorescence microscopy images of wheat leaves: exterior (A) and interior area of the control plant (B), exterior (C) and interior area of the plant treated with fluorescein-labeled liposomes (D). Liposomes are shown in green, and chloroplasts are shown in red.

**Figure 5:**
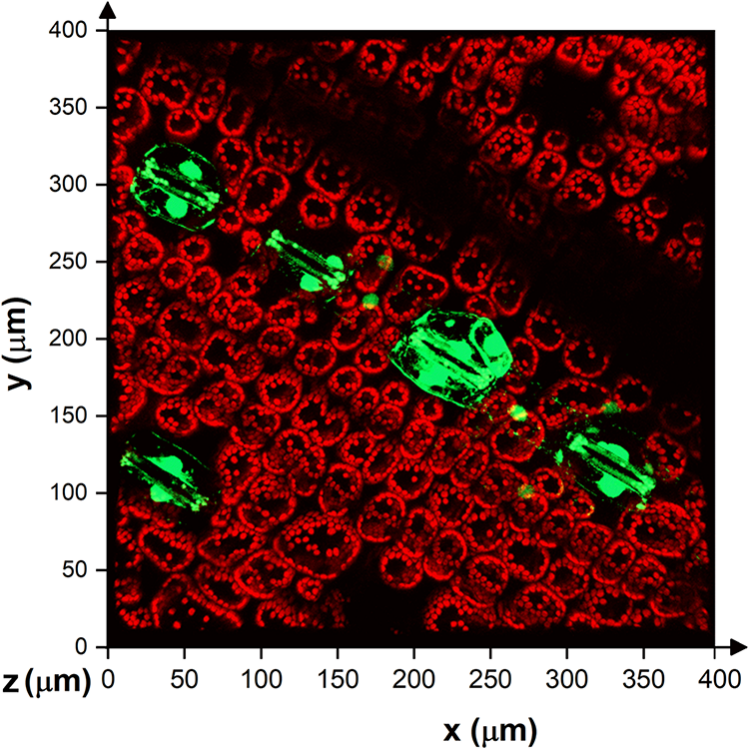
Confocal fluorescence microscopy 3D image of a wheat leaf exposed to fluorescein-labeled liposomes. The analyzed area corresponds to the external part of the leaf.

In wheat leaf, chloroplasts are primarily located within mesophyll cells, close to the intercellular airspaces that connect to stomata, ensuring effective gas exchange and photosynthetic activity. These cells are distributed throughout the leaf tissue and are responsible for capturing light energy and converting it into chemical energy through photosynthesis. Additionally, chloroplasts can also be found in the bundle sheath cells, particularly in C3 plants (C3 photosynthetic pathway) like wheat, where they play a role in photosynthetic processes and metabolic functions.^45,46^

The present results suggest that fluorescein-labeled liposomes were associated with the leaf surface and epidermal tissues and reached the mesophyll tissue beneath the adaxial epidermis during the 24 h period following treatment application, likely involving stomatal-associated pathways; however, cuticular transport cannot be excluded based on the present confocal data. Similar findings were reported by Arsic et al.,^31^ who observed strong signal intensities for the accumulation of ^51^V and ^31^P in the vascular bundles of barley indicating rapid movement of applied ions from the leaf surface to the vasculature within 24 h, as detected by laser ablation-inductively coupled plasma-mass spectrometry (LA-ICP-MS). Both elements also accumulated at points across the epidermis and in the vicinity of stomata, suggesting that these zones may represent potential pathways for ion entry.

Since a longer period (at least 48 h) is generally required for liposomal membrane disruption due to endogenous factors (e.g., cytoplasmic lipases) and osmotic destabilization as suggested by Jahan et al.,^46^ liposome structures may not have disintegrated in our study, giving rise to the observed oval shapes. Once released from liposomes, Se is likely to be transported into the vascular system, located within the mesophyll tissue,^47^ for its distribution and metabolism within the plant.

Confocal microscopy analysis of wheat leaves allowed for the investigation of liposome penetration mechanisms into plant tissues. The results indicate that fluorescein encapsulated within liposomes predominantly accumulates near the stomata, suggesting that these structures serve as the main entry pathway. Once inside the leaf tissue, the liposomes can release their contents into the plant’s vascular system, facilitating intracellular distribution.

This study provides novel insights into the use of nanoencapsulation combined with foliar application as a sustainable strategy for Se biofortification of wheat. Advanced imaging techniques (μ-XRF, μ-XANES and confocal microscopy) revealed the absorption of liposomes through plant surface structures, Se accumulation at the leaf edges, and its transformation into organic forms within 24 h. The slower uptake of encapsulated Se compared to free Se highlights the potential of nanocarriers to control nutrient delivery. These results contribute to a deeper understanding of Se transport mechanisms and establish a basis for future studies. Further research should focus on long-term release studies to better visualize the Se translocation process to other plants tissues and edible parts. The use of cutting-edge research tools such as synchrotron-based μ-XRF and confocal microscopy in plant biofortification studies could significantly enhance the design of nutrient delivery systems and their application contributing to the development of agricultural practices that integrate nanotechnology innovations, leading to methods that are more efficient and environmentally friendly than conventional fertilization strategies.

## Supplementary Material



## Notes

Size distribution by intensity measured using DLS of Se–P90 liposomes (Figure S1); physicochemical properties and Se encapsulated content of in P90H liposomes (Table S1); normalized Se K-edge μ-XANES of plant samples exposed to Se–P90H treatments after 24 h (Figure S2); linear combination fitting analysis (LCF) of the μ-XANES spectra of Se-CK samples (Table S2); K, Mn, Zn, and Se maps in Se-CK (left side) and Se–P90H (right side) cross sections obtained with Dawn software using the RGB mixer tool (Figure S3) (PDF)

## Abbreviations Used

Se: selenium
SeNPs: selenium nanoparticles
μ-XRF: micro-X-ray fluorescence
μ-XANES: micro-X-ray absorption near-edge structure
LHD: layered double hydroxide
P90H: phospholipon 90H
DLS: dynamic light scattering
MES: 2-(*N*-morpholino)­ethanesulfonic acid
Se–P90H: treatment of 1 mM Na_2_SeO_3_ encapsulated in liposomes
Se-CK: control treatment
ICP-MS: inductively coupled plasma mass spectroscopy
LCF: linear combination fitting
SeMet: seleno-l-methionine
SeCys: seleno-l-cystine
MetSeCys: Se-(methyl)­selenocysteine
FDA: fluorescein diacetate
MWCO: molecular weight cut-off
