# Supplementary material for: Tracing Selenium Uptake in Wheat Leaves via Liposome-Mediated Delivery: A Confocal Microscopy and Synchrotron Micro-X-ray Fluorescence Insight
Source: ACS Agric Sci Technol. 2026 Mar 5;6(4):641–9. doi: 10.1021/acsagscitech.5c01076 (PMC13100945; doi:10.1021/acsagscitech.5c01076)
Supplement: Supplementary file 1 [file as5c01076_si_001.pdf]

Supporting information material for
Tracing Selenium Uptake in Wheat Leaves via liposome-Mediated Delivery: A Confocal Microscopy and Synchrotron micro–X-Ray Fluorescence Insight.

Marcia Viltres-Portales[1(¥)], María-Jesús Sánchez-Martín[1*(¥)], Roberto Boada[1], Mercè Llugany[2], Manuel Valiente[1]

[1]GTS Research Group, Department of Chemistry, Faculty of Science, Universitat Autònoma de Barcelona, 08193 Bellaterra, Spain.

[2]Plant Physiology Group (BABVE), Faculty of Biosciences, Universitat Autònoma de Barcelona, 08193, Bellaterra, Spain

(¥) Shared co-first authorship

*Co-first author and author for correspondence: mariajesus.sanchez@uab.cat (ORCiD: 0000-0003-1678-6055)

1. Liposomes characterization

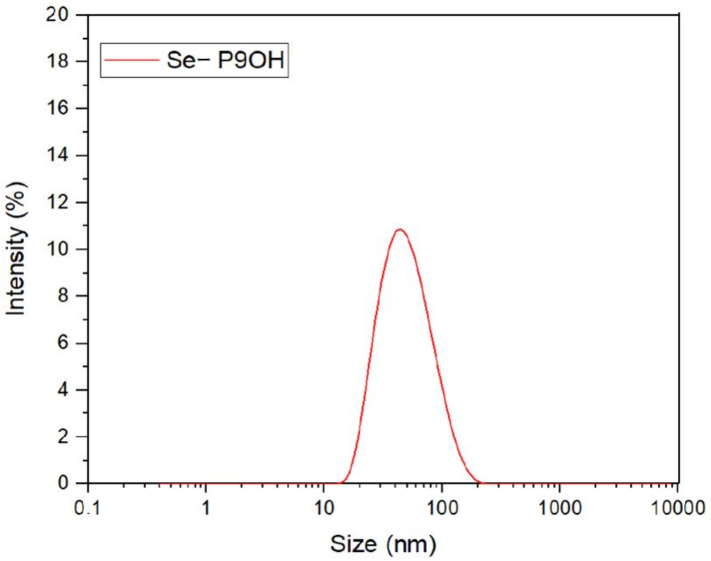

Figure S1. Size distribution by intensity measured using DLS of Se-P90 liposomes

Table S1. Physicochemical properties and Se encapsulated content of in P90H liposomes.

Parameter	Equation	Value
**Z-average (nm)**		46.1 ± 0.9
**PDI**		0.221 ± 0.004
**Z-potential (mV)**		34 ± 2
**Encapsulation Efficacy (%)**	$\left(100 - \frac{mg\ of\ total\ Se - mg\ of\ free\ Se}{mg\ of\ total\ Se}\right) \times 100$	30.2 ± 0.6
**Loading capacity (%)**	$\frac{mg\ of\ encapsulated\ Se}{mg\ of\ initial\ added\ P90H} \times 100$	0.22 ± 0.01

2. uXRF and uXANES

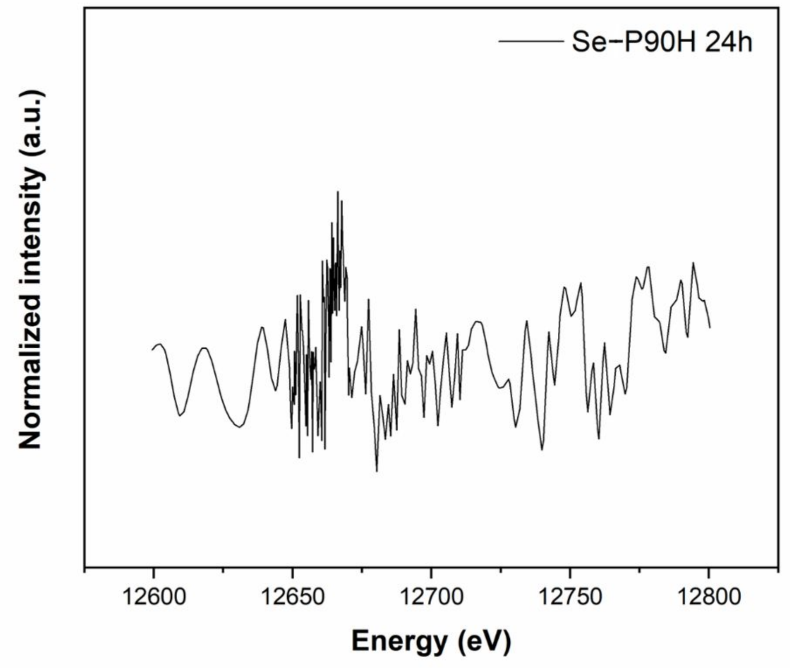

Figure S2. Normalized Se K-edge μ-XANES of plant samples exposed to Se-P90H treatments after 24h.

Table S2. Results from the linear combination fitting analysis (LCF) of the µ-XANES spectra of Se-CK samples

**Relative concentration (%)** SeMet	MetSeCys	SeCys	Se(IV)	Se(VI)	**R-factor**	**Reduced** χ^2
72.6 ± 0.5	11.7 ± 0.7	20.6 ± 0.3	-	-	0.006	0.002

Note: R-factor is a measure of the mean square sum of the misfit at each data point which indicates the goodness of fit. $R - factor = \frac{\sum (data - fit)^2}{\sum data^2}$

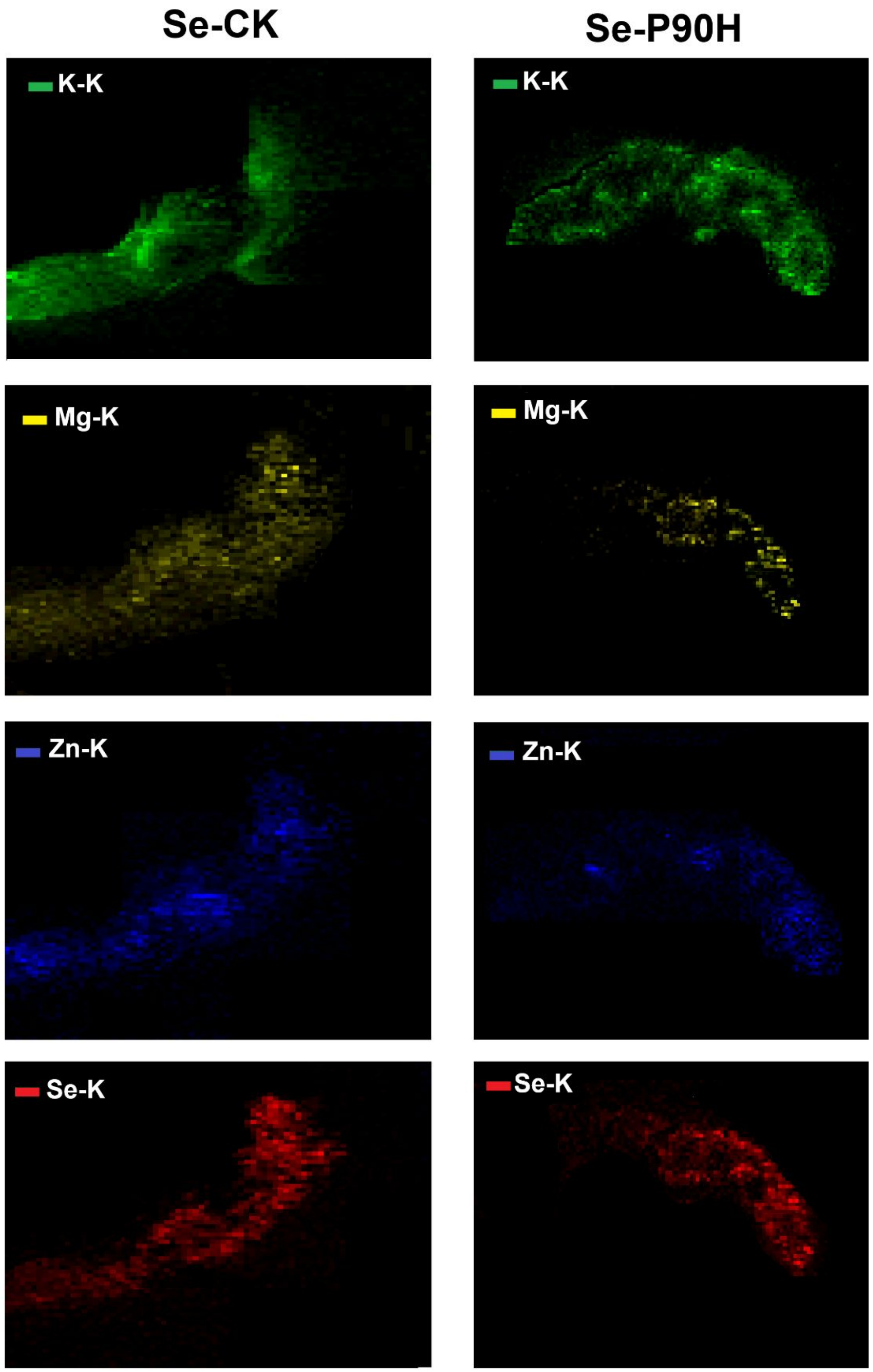

Figure S3. K, Mn, Zn and Se maps in Se-CK (*left side*) and Se-P90H (*right side*) cross sections obtained with Dawn software using the RGB mixer tool.
